# Risk of osteoporotic fracture in a large population-based cohort of patients with rheumatoid arthritis

**DOI:** 10.1186/ar3107

**Published:** 2010-08-03

**Authors:** Seo Young Kim, Sebastian Schneeweiss, Jun Liu, Gregory W Daniel, Chun-Lan Chang, Katie Garneau, Daniel H Solomon

**Affiliations:** 1 Division of Pharmacoepidemiology and Pharmacoeconomics, Department of Medicine, Brigham and Women's Hospital, 75 Francis Street, Boston, MA 02115, USA; 2 Division of Rheumatology, Immunology and Allergy, Department of Medicine, Brigham and Women's Hospital, 75 Francis Street, Boston, MA 02115, USA; 3 HealthCore Inc., 800 Delaware Avenue, Wilmington, DE 19801, USA

## Abstract

**Introduction:**

Although osteoporosis has been reported to be more common in patients with rheumatoid arthritis (RA), little is known whether the risk of osteoporotic fractures in these patients differs by age, sex, and anatomic site.

**Methods:**

A retrospective cohort study was conducted using a health care utilization database. Incidence rates (IRs) and rate ratios (RRs) of osteoporotic fractures with 95% confidence intervals (CIs) were calculated. Multivariable Cox proportional hazards models compared the risk of osteoporotic fracture at typical sites between RA and non-RA patients.

**Results:**

During a median 1.63-year follow-up, 872 (1.9%) of 47,034 RA patients experienced a fracture. The IR for osteoporotic fracture at typical sites among RA patients was 9.6 per 1,000 person-years, 1.5 times higher than the rate of non-RA patients. The IR was highest for hip fracture (3.4 per 1,000 person-years) in RA. The IRs across all age groups were higher for women than men and increased with older age in both groups. The RRs were elevated in RA patients across all common sites of osteoporotic fracture: hip (1.62, 95% CI 1.43 to 1.84), wrist (1.15, 95% CI 1.00 to 1.32), pelvis (2.02, 95% CI 1.77 to 2.30), and humerus (1.51, 95% CI 1.27 to 1.84). After confounding adjustment, a modest increase in risk for fracture was noted with RA (hazard ratio 1.26, 95% CI 1.15 to 1.38).

**Conclusions:**

Our study showed an increased risk of osteoporotic fractures for RA patients across all age groups, sex and various anatomic sites, compared with non-RA patients.

## Introduction

Rheumatoid arthritis (RA) is the most common form of inflammatory arthritis in adults and is characterized by chronic, progressive, systemic inflammation leading to substantial pain, disability, and other morbidities [1-3]. It is well accepted that patients with RA are at an increased risk of osteoporosis and osteoporotic fracture [4-11], even though a previous US population-based study based on the data from the Third National Health and Nutrition Examination Survey (1988 to 1994) did not find a difference in femoral neck bone mineral density (BMD) between RA and non-RA patients [12].

Osteoporosis, particularly in patients with RA, is a multifactorial condition. Some studies have suggested the association between osteoporosis and proinflammatory cytokines such as TNF-α, IL-1 and IL-6, as these cytokines play an important role in bone resorption [13-19]. Positive correlations between osteoporosis and C-reactive protein (CRP), a marker of active inflammation, have been observed, although not always confirmed, in a number of epidemiologic studies [16,17,20-22]. Other known risk factors for osteoporosis include older age, female sex, menopause, lower body mass index, glucocorticoids use, high RA disease activity, long RA disease duration, and decreased physical activity [8,9,19,22-27]. Osteoporotic fracture, particularly at the hips, is associated with the risk of falling [28,29]. Fall-related risk factors such as impaired heel-toe walking and inability to do stand-ups without arm use [30] were more common in patients with RA than non-RA patients [24], probably related to impaired balance and poor lower limb muscle strength. In addition, RA patients have chronic polyarticular pain, which increases a risk of falls [31]. A high prevalence of osteoporosis is observed as 50% of 925 female RA patients in a large Italian multicenter cross-sectional study had osteoporosis defined as BMD T-score lower than -2.5 in at least one region of measurement, although it might have been overestimated due to referral bias [23].

Although an increased risk of osteoporosis in RA patients is well-reported, little information is available with regard to the population-based frequency of incident osteoporotic fractures in RA patients and their risks relative to different age groups, sex, anatomic site, and glucocorticoid use. We studied a very large cohort from a health care utilization database: to estimate the incidence rate (IR) of typical osteoporotic fractures among RA patients relative to age, sex and anatomic site; to assess the risk of typical osteoporotic fractures among RA compared with non-RA patients; and to evaluate the effects of rheumatoid factor (RF) and acute phase reactants on fracture risk among patients with RA.

## Materials and methods

### Data source

We conducted a cohort study using the administrative claims data in the HealthCore Integrated Research Database (HIRD) for the period 1 January, 2001 to 30 June, 2008. This database contained longitudinal claims information including medical diagnoses, procedures, hospitalizations, physician visits, and pharmacy dispensings on more than 28 million fully-insured subscribers, with medical and pharmacy coverage, to 14 Blue Cross/Blue Shield health plans across the USA. Results for outpatient laboratory tests, including CRP, erythrocyte sedimentation rate (ESR), and RF were available on a subset of beneficiaries. No information was available on the results of BMD tests or on other radiologic procedures.

Personal identifiers were removed from the dataset before the analysis to protect subject confidentiality. Patient informed consent was, therefore, not required. This study was approved by Brigham and Women's Hospital's Institutional Review Board and Data Use Agreements were in place with HealthCore, Inc.

### Study cohort

Adults aged 18 years or older with at least two visits for RA identified with the *International Classification of Diseases*, Ninth Revision, Clinical Modification (*ICD *9-CM) code, 714.xx, were eligible for this study. Subjects who did not have a diagnosis of RA at any time during the entire study period were eligible to be part of the non-RA cohort. From this eligible non-RA cohort, five patients, matched on age and sex were selected for every subject with RA. The follow-up period began at the index date, defined as the date of second RA diagnosis for RA patients and the date of the first medical claim for the non-RA patients. We further required all subjects to have at least 12 months of continuous health plan eligibility before the start of follow-up. Subjects were then followed until occurrence of outcomes, loss of eligibility, end of study database, or death.

### Outcome definitions

The definitions of fracture were based on diagnoses and procedure codes contained within the study database [see Additional file 1]. We included hip, wrist, humerus, and pelvis fractures, because these are considered to be typical sites of osteoporotic fracture and can be accurately defined in administrative claims databases [32]. A composite of fractures at these four sites ('any osteoporotic fracture') was also considered. Patients were censored at their first fracture in the 'any osteoporotic fracture' analysis.

### Covariates

Variables potentially related to a future fracture were assessed using the data from the 12 months prior to the index date. These variables included demographic factors (age and sex), osteoporosis-related factors (osteoporosis diagnosis, osteoporosis medications, prior fracture, BMD test, Parkinson's disease, Alzheimer's disease, prior falls, and other comorbidities), use of other medications likely to be associated with bone metabolism or fall risks (oral glucocorticoids, anticonvulsants, benzodiazepines, selective serotonin reuptake inhibitors (SSRIs), beta blockers, proton pump inhibitors, and opioids), and health care utilization factors (number of physician visits, acute care hospitalizations, and number of different medications). To quantify patients' comorbidities, we calculated the Deyo-adapted Charlson comorbidity index based on *ICD*-9-CM [33,34]. The comorbidity index is a summative score, based on 19 major medical conditions including myocardial infarction, pulmonary disease, renal disease, hepatic disease, diabetes, cancer, HIV infection, etc. A score of 0 represents absence of comorbidity and a higher score indicates a greater number of comorbid conditions.

To explore a potential association between the disease status and fracture risk in patients with RA, we calculated the Claims-based Index for RA Severity (CIRAS) scores [35], based on age, sex, number of tests for inflammatory markers, number of chemistry panels and platelet counts ordered, RF, the number of rehabilitation and rheumatology visits, and Felty's syndrome diagnosis, and examined outpatient laboratory data such as acute phase reactants (i.e., ESR or CRP) and RF levels in a subgroup of the RA cohort.

### Statistical analyses

We compared the baseline characteristics between the RA and non-RA cohorts. Fracture IRs with 95% confidence intervals (CI) were calculated for all patients, and then stratified by age and sex. Rate ratios (RRs) were estimated by dividing the IR among RA patients by the IR among non-RA [36]. Similar analyses were carried out for specific anatomic site fractures, and then stratified by baseline oral glucocorticoid use. Finally, to adjust for potential confounders, separate Cox proportional hazard models were used to compare the risks for any fracture and fracture at each site among RA patients with those in non-RA patients. Additional Cox proportional hazard models focused on the risks relative to age and sex. Finally, we conducted subgroup analyses to examine whether positive RF and elevated acute phase reactants, either ESR or CRP, increased a risk of fracture in RA patients. All analyses were performed using SAS 9.1 Statistical Software (SAS Institute Inc., Cary, NC, USA).

## Results

### Cohort selection

**Figure 1:**
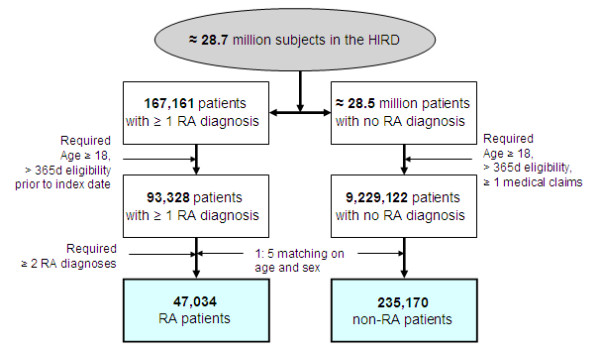
**Selection of the study cohort**. d, days; HIRD, HealthCore Integrated Research Database; RA, rheumatoid arthritis.

There were more than 28.7 million potentially eligible subjects in the study database. Figure 1 displays our cohort selection process. There were initially 167,161 subjects with at least one RA diagnosis and approximately 28.5 million subjects with no RA diagnosis at any time during the entire study period. Subsequently, 93,328 patients with at least one RA diagnosis, representing 0.32% of the potentially eligible population, and 9.2 million subjects with no RA diagnosis at any time during the study period met our eligibility criteria. We then matched 92,827 RA patients to 921,715 non-RA subjects by age, sex, plan type, calendar year, and state with a 1:10 ratio. After requiring a minimum of 12-months of eligibility prior to two physician visits for RA, the number of RA patients dropped to 47,034. Our final study cohort includes 47,034 RA patients and 235,170 non-RA patients matched on age and sex with a 1:5 ratio. The median follow-up time was 1.63 years for RA patients and 1.64 years for non-RA patients, accounting for 91,315 person-years in RA subjects and 488,929 person-years in non-RA patients.

### Patient characteristics

Baseline characteristics of the age- and sex-matched cohorts were compared (Table 1). The median age was 55 years and 73% were women in both cohorts. Substantial differences across almost all other baseline characteristics were observed between the cohorts, with the prevalence of fracture risk factors much more common in RA patients than non-RA subjects. A recorded diagnosis of osteoporosis, comorbidity, oral glucocorticoid use, and health care utilization including physician visits and hospitalization were more commonly noted in patients with RA.

**Table 1:** Baseline characteristics of the study cohort 12 months prior to the index date

	RA patients (*n *= 47,034)	**Non-RA patients**^**§**^ (*n *= 235,170)
**Demographic**		
Age, years	55 (46-64)	55 (46-64)
Sex, female	34,169 (73)	170,845 (73)
**Osteoporosis-related**		
Osteoporosis diagnosis	8,385 (18)	16,724 (7)
Osteoporosis medication	5,634 (12)	14,067 (6)
Prior fracture	413 (0.9)	1,322 (0.6)
Bone mineral density test	5,802 (12)	13,307 (6)
Parkinson's disease	177 (0.4)	718 (0.3)
Alzheimer's disease	156 (0.3)	752 (0.3)
Prior fall	609 (1)	2351 (1)
At least one comorbidity	39,259 (83)	53,299 (23)
Comorbidity index *	1 (1-2)	0 (0-0)
CIRAS score	5.2 (3.7-6.9)	3.6 (2.9-4.3)
**Medication use**		
Oral glucocorticoids	18,222 (39)	15,342 (7)
Anticonvulsants	3,703 (8)	7,795 (3)
Benzodiazepines	8,362 (18)	25,754 (11)
SSRIs	7,289 (16)	22,920 (10)
Beta-blockers	6,520 (14)	25,166 (11)
Proton pump inhibitors	9,415 (20)	19,859 (8)
Opioids	22,049 (47)	54,204 (23)
**Health care utilization**		
Number of physician visits	8 (5-14)	2 (0-6)
Hospitalizations	8,894 (19)	25,131(11)
Number of all prescription drugs	8 (4-13)	3 (0-7)

Data are expressed as number (%) or median (interquartile range); ^§ ^1:5 matched on age and sex. *, Charlson-Deyo Comorbidity Index [33]; a score of 0 represents absence of comorbidity and higher scores indicate a greater burden of comorbidity.

CIRAS, Claims-based Index for RA Severity; RA, rheumatoid arthritis; SSRI, selective serotonin reuptake inhibitor.

### Incidence rates of any fracture

During the study follow-up, 3,968 patients (1.4%) of the study population experienced a fracture. As shown in Table 2, the IR of fracture at any of the four sites (wrist, humerus, hip, and pelvis) among RA patients was 9.6 per 1,000 person-years and 1.5 times higher than that of non-RA patients (6.3 per 1,000 person-years). The RRs of experiencing any osteoporotic fractures among RA patients compared with non-RA ranged from 1.35 (above age 85 years) to 2.13 (age between 65 and 74 years). Similar age trends were observed in the stratified analyses by sex.

**Table 2:** Incidence rates of any osteoporotic fractures* for study population, by age and sex

Age (years)	RA patients			**Non-RA patients**^**§**^			
			
	Fractures, N	Person-years	IR (95% CI)	Fractures, N	Person-years	IR (95% CI)	**RR **^**† **^**(95% CI)**
**All**							
All ages	872	91,315	9.6 (8.9-10.2)	3,096	488,929	6.3 (6.1-6.6)	1.51 (1.40-1.63)
< 50	92	31,458	2.9 (2.3-3.5)	285	164,629	1.7 (1.5-1.9)	1.69 (1.34-2.14)
50-64	225	39,376	5.7 (5.0-6.5)	769	203,945	3.8 (3.5-4.0)	1.51 (1.30-1.75)
65-74	186	11,278	16.5 (14.1-18.9)	504	65,018	7.8 (7.1-8.4)	2.13 (1.80-2.52)
75-84	236	7,043	33.5 (29.2-37.8)	900	41,385	21.8 (20.3-23.2)	1.54 (1.33-1.78)
85+	133	2,160	61.6 (51.1-72.1)	638	13,952	45.7 (42.2-49.3)	1.35 (1.12-1.63)

**Women**							
All ages	742	66,785	11.1 (10.3-11.9)	2,653	355,094	7.5 (7.2-7.8)	1.49 (1.37-1.62)
< 50	73	23,267	3.1 (2.4-3.9)	197	121,361	1.6 (1.4-1.9)	1.94 (1.48-2.54)
50-64	180	28,631	6.3 (5.4-7.2)	652	145,980	4.5 (4.1-4.8)	1.41 (1.20-1.66)
65-74	166	8,006	20.7 (17.6-24)	436	45,729	9.5 (8.6-10.4)	2.18 (1.82-2.61)
75-84	204	5,153	39.6 (34.2-45)	780	30,532	25.6 (23.8-27.3)	1.55 (1.33-1.81)
85+	119	1,768	67.3 (55.2-79.4)	588	11,492	51.2 (47.0-55.3)	1.32 (1.08-1.61)

**Men**							
All ages	130	24,530	5.3 (4.4-6.2)	443	133,835	3.3 (3.0-3.6)	1.60 (1.32-1.95)
< 50	19	8,231	2.3 (1.3-3.4)	88	43,268	2.0 (1.6-2.5)	1.14 (0.69-1.87)
50-64	45	10,745	4.2 (3.0-5.4)	117	57,965	2.0 (1.7-2.4)	2.07 (1.47-2.92)
65-74	20	3,272	6.1 (3.4-8.8)	68	19,289	3.5 (2.7-4.4)	1.73 (1.05-2.85)
75-84	32	1,890	16.9 (11.1-22.8)	120	10,853	11.1 (9.1-13.0)	1.53 (1.04-2.26)
85+	14	392	35.8 (17.0-54.5)	50	2,460	20.3 (14.7-26.0)	1.76 (0.97-3.18)

Rates are shown per 1,000 person-years.

^§^, 1:5 matched on age and sex; *, includes incident fractures of hip, wrist, pelvis, or humerus; ^†^, unadjusted. CI, confidence interval; IR, incidence rates;. RR, rate ratio.

### Incidence rates of fracture by anatomic sites

Site-specific fracture IRs were calculated for hip, wrist, humerus, and pelvis (Table 3). Among the RA patients, humerus fracture had the lowest IR (1.6 per 1,000 person-years) and hip fracture the highest (3.4 per 1,000 person-years). The IR for humerus fracture was also lowest (1.0 per 1,000 person-years) among non-RA patients, but the IR for wrist fracture was the highest (2.2 per 1,000 person-years). Among women with RA, the highest IR was noted for pelvis fracture (4.0 per 1,000 person-years). The fracture IR at hip was 3.8 per 1,000 person-years. In male RA patients, the fracture IR was 2.4 per 1,000 person-years at hip and 1.5 per 1,000 person-years at pelvis. The RRs were elevated across all anatomic sites for both men and women, ranging from 1.12 to 2.05, except those for wrist fracture for both men and women, and humerus fracture for men.

**Table 3:** Incidence rates of fractures for study population, by anatomic site and sex

Anatomic site	RA patients			**Non-RA patients **^**§**^			
			
	Fractures, n	Person-years	IR (95% CI)	Fractures, n	Person-years	IR (95% CI)	**RR **^**†**^ (95% CI)
**All**							
Wrist	234	92,167	2.5 (2.2-2.9)	1,088	492,138	2.2 (2.1-2.3)	1.15 (1.0-1.32)
Humerus	143	92,326	1.6 (1.3-1.8)	498	493,117	1.0 (0.9-1.1)	1.53 (1.27-1.84)
Hip	311	92,161	3.4 (3-3.7)	1,027	492,569	2.1 (2-2.2)	1.62 (1.43-1.84)
Pelvis	304	92,100	3.3 (2.9-5.7)	804	492,735	1.6 (1.5-1.7)	2.02 (1.77-2.30)
Any site	872	91,314	9.6 (8.9-10.2)	3,096	488,929	6.3 (6.1-6.6)	1.51 (1.40-1.63)

**Women**							
Wrist	197	67,533	2.9 (2.5-3.3)	933	357,899	2.6 (2.4-2.8)	1.12 (0.96-1.31)
Humerus	130	67,646	1.9 (1.6-2.3)	432	358,719	1.2 (1.1-1.3)	1.60 (1.32-1.95)
Hip	253	67,535	3.8 (3.3-4.2)	873	358,252	2.4 (2.3-2.6)	1.54 (1.34-1.77)
Pelvis	267	67,449	4.0 (3.5-4.4)	700	358,388	2.0 (1.8-2.1)	2.03 (1.76-2.34)
Any site	742	66,785	11.1 (10.3-11.9)	2,653	355,094	7.5 (7.2-7.8)	1.49 (1.37-1.62)

**Men**							
Wrist	37	24,634	1.5 (1.0-2.0)	155	134,239	1.2 (1.0-1.3)	1.30 (0.91-1.86)
Humerus	13	24,680	0.5 (0.2-0.8)	66	134,398	0.5 (0.4-0.6)	1.08 (0.60-1.96)
Hip	58	24,626	2.4 (1.8-3.0)	154	134,317	1.2 (1.0-1.3)	2.05 (1.52-2.77)
Pelvis	37	24,651	1.5 (1.0-2.0)	104	134,346	0.8 (0.6-0.9)	1.95 (1.34-2.84)
Any site	130	24,530	5.3 (4.4-6.2)	443	133,835	3.3 (3.0-3.6)	1.60 (1.32-1.95)

Rates are shown per 1,000 person-years. ^†^, unadjusted. ^§^, 1:5 matched on age and sex. CI, confidence interval; IR, incidence rates;. RR, rate ratio.

### Adjusted risks of fracture among patients with RA

**Figure 2:**
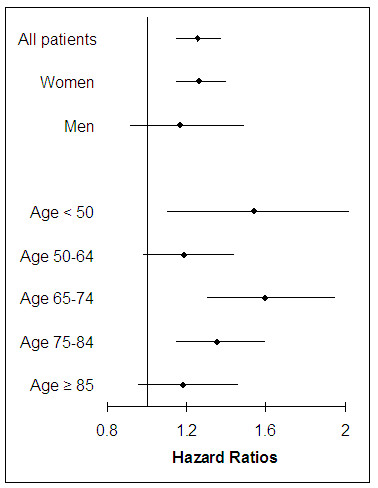
**Adjusted hazard ratios with 95% confidence intervals for fractures among RA patients compared with non-RA patients, by age and sex**. Simultaneously adjusted for age, sex, medications including oral glucocorticoids, osteoporosis drugs, beta blockers, opioids, anticonvulsants, proton pump inhibitors, and selective serotonin reuptake inhibitors, Parkinson's disease, Alzheimer's disease, prior fall, prior fracture, history of bone mineral density test, comorbidity index, Claims-based Index for RA Severity score, and other health care utilization characteristics.

**Figure 3:**
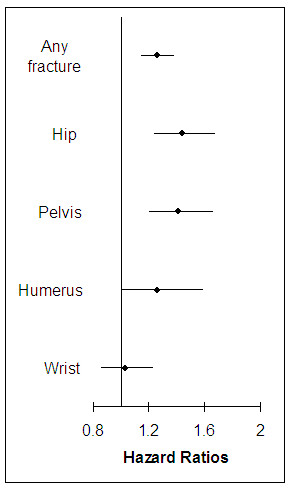
**Adjusted hazard ratios with 95% confidence intervals for fracture among RA patients compared with non-RA patients by anatomic site**. Simultaneously adjusted for age, sex, medications including oral glucocorticoids, osteoporosis drugs, beta blockers, opioids, anticonvulsants, proton pump inhibitors, and selective serotonin reuptake inhibitors, Parkinson's disease, Alzheimer's disease, prior fall, prior fracture, history of bone mineral density test, comorbidity index, Claims-based Index for RA Severity score, and other health care utilization characteristics.

All the variables listed in Table 1 were adjusted by fitting multivariable Cox proportional hazards models. The adjusted hazard ratio (HR) for any fracture was 1.26 (95% CI 1.15 to 1.38) in RA patients compared with non-RA. Age, female sex, osteoporosis drugs, SSRIs, anticonvulsants, and opioids, history of Parkinson's disease, prior fall and fracture, and hospitalization, numbers of physician visits and prescription drugs, and the comorbidity index were independently associated with an increased risk of fracture [see Additional file 2]. Prior use of oral glucocorticoids also increased a risk of osteoporotic fracture (HR 1.15, 95% CI 1.03 to 1.27). Furthermore, the adjusted HRs were consistently elevated in RA patients across all age and sex groups (Figure 2). Additional multivariable Cox regression analyses (Figure 3) showed increased HRs associated with RA for fractures at the hip (1.44, 95% CI 1.24 to 1.67) and pelvis (1.41, 95% CI 1.20 to 1.66), but not for humerus (1.26, 95% CI 1.00 to 1.58) or wrist fractures (1.03, 95% CI 0.86 to 1.23).

### Subgroup analyses on laboratory data

Subgroup analyses examined the effect of RF (*n *= 7,016, 15%) and acute phase reactants (*n *= 10,309, 22%) on fracture risk among the RA patients with these laboratory data available. Positive RF (adjusted HR, 1.39, 95% CI 0.78 to 2.49) and elevated acute phase reactants (adjusted HR 1.30, 95% CI 0.85 to 1.99) were associated, although not statistically significant, with an increased fracture risk among the patients with RA after multivariable adjustment in Cox models. Prior use of oral glucocorticoids also increased risk of osteoporotic fracture among the patients in this subgroup analysis (adjusted HR 1.36, 95% CI 0.86 to 2.14).

## Discussion

Osteoporotic fractures are more common in patients with RA than the general population. We studied the IRs of osteoporotic fracture of the humerus, wrist, hip, and pelvis in a cohort of 47,034 RA patients using claims data. The IRs of fracture were 1.5 times higher among patients with RA compared with non-RA patients regardless of age, sex, or anatomic sites. This association between RA and fracture weakened after multivariable adjustment for known risk factors of osteoporosis including oral glucocorticoid use.

Chronic inflammation has been recently recognized as a potential risk factor for osteoporosis and fracture [14]. The Health Aging and Body Composition Study showed that elevated inflammatory markers, such as IL-2, IL-6, CRP, and TNF-α, were associated with osteoporotic fracture [18]. In a study of 74 post-menopausal women with RA, high disease activity, measured by high CRP levels and ESR, and an elevated IL-6 were associated with an increase in periarticular as well as systemic bone resorption [19]. We performed subgroup analyses adjusted for either RF or acute phase reactants, in which the activity of RA or inflammation can be better, but not completely, taken into consideration. Both RF positivity and elevated acute phase reactant levels increased the risk of fracture but the association was not statistically significant. The association between oral glucocorticoid use and fracture risk remained increased among the subgroup of RA patients in whom these laboratory data were available. Nonetheless, our subgroup analyses might not have sufficient power to assess the independent association between these potential risk factors (i.e., RF, acute phase reactants, oral glucocorticoid use, and other medications) and fracture risks.

The recently developed World Health Organization fracture risk assessment tool, FRAX^®^, computes the 10-year probability of hip fracture or a major osteoporotic fracture, based on individual patient models that integrate the risks associated with BMD at the femoral neck as well as clinical risk factors such as age, sex, smoking status, use of glucocorticoids, history of osteoporosis and prior fall, and RA [37]. Fracture risk related to RA independent of BMD (RR 1.73, 95% CI 0.94 to 3.20) incorporated in this tool is higher than our results (HR 1.26, 95% CI 1.15 to 1.38) [38]. This difference is probably associated with baseline characteristics of study population and degree of confounding adjustment.

Several strengths of this study are worth noting. We examined a very large cohort of RA and non-RA patients in a population that is representative of the US commercially-insured population. Our multivariable Cox models were simultaneously adjusted for more than 20 risk factors of osteoporotic fracture. Various subgroup analyses enabled us to provide specific risks relative to age, sex, anatomic site, and laboratory results. Our results are consistent with a population-based study of 30,262 RA patients in the UK [9]. The authors noted that patients with RA had an increased risk of fracture compared with the non-RA patients (RR 1.5, 95% CI 1.4 to 1.6). Similar to our findings, the RR of fracture was highest for hip fracture and lowest for wrist.

There are, however, limitations to our study. First, this cohort study is likely to be subject to residual confounding by race, body mass index, calcium and vitamin D intake, frailty, and other unmeasured risk factors. Although we assessed variables potentially related to a future fracture using the data from the 12 months prior to the index date, this time period might not be long enough to capture all the information on potential confounders. We used both the comorbidity index and CIRAS scores to minimize the effect of such confounders. The comorbidity index has been widely used to measure comorbidity in various medical fields since its development [33,39-41]. Previous research showed moderate correlations between the CIRAS and a previously validated medical records-based index of severity [35]. The substantial change in point estimates after multivariable adjustment indicates that further improved adjustment may explain our findings. We also conducted additional analyses on a subgroup of RA patients, in whom laboratory data were available, to assess whether the severity of RA affects the risk of fracture, and observed an increased risk associated with positive RF and elevated acute phase reactants, although it was not statistically significant. The analyses of laboratory test results need to be interpreted with caution as ordering laboratory tests in clinical practice is not a random process but often related to the disease status. Second, there could be misclassification with the diagnoses of RA and osteoporotic fractures as we mainly relied on diagnosis and procedure codes to identify them. Both the *ICD *codes for RA and the *ICD *codes and/or procedure codes for fractures have been used in a number of studies [32,42-45]. Third, we relied on prescription dispensing records in the database to determine patients' drug exposures including oral glucocorticoids. It may not be the most accurate way to verify individuals' daily drug exposures, but it is still considered as one of the best ways to ascertain drug exposure status in non-experimental settings [46].

Finally, as true in most epidemiologic studies, patients were not randomly exposed to drugs in our study. Therefore, we cannot exclude the possibility of confounding by indication with regard to the effect of glucocorticoids on fracture risk in patients with RA. The detrimental effect of glucocorticoids on fracture might have been confounded if the drug was selectively given to patients with a higher degree of systemic inflammation and RA severity, and if the degree of systemic inflammation and the severity of RA correlated with the risk of fracture. Several studies recently reported a potentially beneficial effect of low-dose, short-term systemic glucocorticoids on BMD in RA due to its anti-inflammatory effects [25,47-49]. Although this study was not designed to address a potential role of disease-modifying anti-rheumatic drugs including TNF-α inhibitors in the risk of osteoporotic fracture among RA patients, there is some evidence suggesting a beneficial effect of such drugs on bone loss [15,48,50]. The exact effects of glucocorticoids or disease-modifying anti-rheumatic drugs on the risk of fractures in RA should be further studied.

## Conclusions

Our study found that patients with RA are at an increased risk of osteoporotic fractures across age groups, sex and various anatomic sites. An independent association between the use of oral glucocorticoids and fracture risk was confirmed. Future research that evaluates the effect of RA treatments on the risk of osteoporosis would be important.

## Abbreviations

BMD: bone mineral density; CI: confidence interval; CIRAS: Claims-based Index for RA Severity; CRP: C-reactive protein; ESR: erythrocyte sedimentation rate; HIRD: HealthCore Integrated Research Database; HR: hazard ratio; *ICD *9-CM: *International Classification of Diseases*, Ninth Revision, Clinical Modification; IR: incidence rate; RA: rheumatoid arthritis; RF: rheumatoid factor; RR: rate ratios; TNF: tumor necrosis factor.

## Competing interests

SY Kim, J Liu, GW Daniel, C-L Chang, and K Garneau declare that they have no competing interests. S Schneeweiss received research grants from Pfizer, Inc. DH Solomon received research grants from Abbot, BMS, Merck & Co., Inc. Novartis, and Amgen, Inc.

## Authors' contributions

All authors participated in the study conception. SYK, SS, and DHS participated in the study design. SYK, SS, GWD, C-LC, KG, and DHS participated in data acquisition. SYS, SS, JL, and DHS participated in data analysis and interpretation. All authors participated in manuscript preparation and revision. All authors read and approved the final manuscript.

## Supplementary Material

Additional file 1 **Definition of fracture outcomes**. A list of diagnosis and procedure codes to define fracture outcomes. Click here for file

Additional file 2 **Adjusted hazard ratios with 95% confidence intervals for any osteoporotic fractures**. Adjusted hazard ratios (HR) with 95% confidence intervals (CI) for any osteoporotic fracture from a multivariable Cox proportional hazards model. Click here for file
