# Supplementary material for: Risk of osteoporotic fracture in a large population-based cohort of patients with rheumatoid arthritis
Source: Arthritis Res Ther. 2010 Aug 3;12(4):R154. doi: 10.1186/ar3107 (PMC2945054; doi:10.1186/ar3107)
Supplement: Additional file 1 — Definition of fracture outcomes. A list of diagnosis and procedure codes to define fracture outcomes. [file ar3107-S1.DOC]

**Appendix 1.** Definition of fracture outcomes

| Fracture | Definition |
| --- | --- |
| Humerus | Humerus fracture diagnosis (ICD-9: 812.xx, 733.11) AND procedure within 30 days of fracture date (ICD-9: 78.52, 79.01, 79.11, 79.21, 79.31, 79.61; CPT-4: 23600, 23605, 23610, 23615, 23620, 23625, 23630, 23665, 23670, 23680, 24500, 24505, 24506, 24510, 24515, 24530, 24531, 24535, 24536, 24538, 24540, 24542, 24545, 24560, 24565, 24570, 24575, 24581, 24583, 24585-8, 24516) |
| Wrist | Radius/ulna fracture diagnosis (ICD-9: 813.xx, 733.12) AND procedure within 30 days of fracture date (ICD-9: 78.53, 79.02, 79.12, 79.22, 79.32, 79.62; CPT-4: 24620, 24625, 24635, 24650, 24655, 24660, 24665-6, 24670, 24680, 24685, 25500, 25505, 25510, 25515, 25530, 25535, 25540, 25545, 25560, 25565, 25570, 25575, 25600, 25605, 25610-1, 25615, 25620, 25650) |
| Hip | Hip fracture diagnosis (ICD-9 code: 820.xx,733.14) during hospitalization AND procedure code during hospitalization (ICD-9: 78.55, 79.05, 79.15, 79.25, 79.35, 79.65; CPT-4: 27230-27248) |
| Pelvis | Pelvis fracture diagnosis (ICD-9: 808.xx) |

### ICD-9: [International Classification of Diseases](http://www.who.int/whosis/icd10/) -9; CPT-4: [Current Procedural Terminology](http://ama-assn.org/ama/pub/category/3113.html) -4
