# Supplementary material for: Risk of osteoporotic fracture in a large population-based cohort of patients with rheumatoid arthritis
Source: Arthritis Res Ther. 2010 Aug 3;12(4):R154. doi: 10.1186/ar3107 (PMC2945054; doi:10.1186/ar3107)
Supplement: Additional file 2 — Adjusted hazard ratios with 95% confidence intervals for any osteoporotic fractures. Adjusted hazard ratios (HR) with 95% confidence intervals (CI) for any osteoporotic fracture from a multivariable Cox proportional hazards model. [file ar3107-S2.DOC]

**Appendix 2.** Adjusted hazard ratios (HR) with 95% confidence intervals (CI) for any osteoporotic fractures.

| Variables | **Any osteoporotic fracture** | |
| --- | --- | --- |
|  | HR | 95% CI |
| Rheumatoid arthritis | 1.26 | 1.15-1.38 |
| Age, year * | 1.06 | 1.06-1.07 |
| Gender, female | 1.89 | 1.73-2.07 |
| Osteoporosis drugs | 1.29 | 1.18-1.41 |
| Proton pump inhibitors | 1.00 | 0.90-1.10 |
| SSRIs | 1.22 | 1.11-1.35 |
| Anticonvulsants | 1.28 | 1.12-1.46 |
| Beta-blockers | 0.92 | 0.84-1.01 |
| Opioids | 1.15 | 1.06-1.24 |
| Benzodiazepines | 1.09 | 0.99-1.20 |
| Oral glucocorticoids | 1.15 | 1.03-1.27 |
| Alzheimer’s disease | 1.09 | 0.84-1.40 |
| BMD test | 0.93 | 0.84-1.03 |
| Parkinson’s disease | 1.61 | 1.26-2.06 |
| Prior fracture | 5.97 | 5.29-6.75 |
| Prior fall | 1.44 | 1.24-1.68 |
| Hospitalization | 1.13 | 1.04-1.23 |
| Number of physician visit * | 1.01 | 1.01-1.02 |
| Number of all prescription drugs * | 0.99 | 0.98-1.00 |
| CIRAS score * | 0.99 | 0.95-1.03 |
| Comorbidity index * | 1.05 | 1.03-1.08 |

Simultaneously adjusted for age, gender, use of oral glucocorticoids and osteoporosis drugs, other medications such as beta blockers, opioids, anticonvulsants, proton pump inhibitors, and selective serotonin re-uptake inhibitors, presence of Parkinson’s disease and Alzheimer’s disease, prior fall, prior fracture, history of bone mineral density (BMD) test, Comorbidity Index, CIRAS score, and other health care utilization characteristics; SSRIs: selective serotonin reuptake inhibitors; *, hazard ratio for any osteoporotic fracture increases for every 1 unit (e.g., number or score) of the continuous variables.
